# A restatement of the natural science evidence base concerning grassland management, grazing livestock and soil carbon storage

**DOI:** 10.1098/rspb.2023.2669

**Published:** 2024-01-24

**Authors:** Matthew W. Jordon, Jean-Charles Buffet, Jennifer A. J. Dungait, Marcelo V. Galdos, Tara Garnett, Michael R. F. Lee, John Lynch, Elin Röös, Timothy D. Searchinger, Pete Smith, H. Charles J. Godfray

**Affiliations:** ^1^ Oxford Martin School, University of Oxford, 34 Broad Street, Oxford OX1 3BD, UK; ^2^ School of Geography, College of Life and Environmental Sciences, University of Exeter, Stoker Road, Exeter EX4 4PY, UK; ^3^ Carbon Management Centre, Scotland's Rural College (SRUC), Peter Wilson Building, The King's Buildings, West Mains Road, Edinburgh EH9 3JG, UK; ^4^ Rothamsted Research, West Common, Harpenden, Hertfordshire AL5 2JQ, UK; ^5^ TABLE, Environment Change Institute, University of Oxford, 3 South Parks Road, Oxford OX1 3QY, UK; ^6^ Harper Adams University, Newport TF10 8NB, UK; ^7^ Nature-Based Solutions Initiative, Department of Biology, University of Oxford, 11a Mansfield Road, OX1 3SZ, UK; ^8^ Swedish University of Agriculture Sciences, Ulls hus, Almas allé 8, Uppsala, SE-750 07, Sweden; ^9^ School of Public and International Affairs, Princeton University, 318 Robertson Hall, NJ 08544-1013, USA; ^10^ Institute of Biological and Environmental Sciences, University of Aberdeen, 23 St Machar Drive, Aberdeen AB24 3UU, UK; ^11^ Department of Biology, University of Oxford, 11a Mansfield Road, Oxford OX1 3SZ, UK

**Keywords:** pasture, soil organic matter, carbon sequestration, ruminants, greenhouse gas emissions, review

## Abstract

Approximately a third of all annual greenhouse gas emissions globally are directly or indirectly associated with the food system, and over a half of these are linked to livestock production. In temperate oceanic regions, such as the UK, most meat and dairy is produced in extensive systems based on pasture. There is much interest in the extent to which such grassland may be able to sequester and store more carbon to partially or completely mitigate other greenhouse gas emissions in the system. However, answering this question is difficult due to context-specificity and a complex and sometimes inconsistent evidence base. This paper describes a project that set out to summarize the natural science evidence base relevant to grassland management, grazing livestock and soil carbon storage potential in as policy-neutral terms as possible. It is based on expert appraisal of a systematically assembled evidence base, followed by a wide stakeholders engagement. A series of evidence statements (in the appendix of this paper) are listed and categorized according to the nature of the underlying information, and an annotated bibliography is provided in the electronic supplementary material.

## 1. Introduction

Approximately 50% of the UK's land area is managed as pasture or grassland used for ruminant livestock production [1]. The equivalent global figure is about 25% [2]. The extent of such grassland, and how it is managed, is very significant for national and global greenhouse gas fluxes, and hence climate change. Pasture and grasslands contain substantial stocks of carbon, though can also act as carbon sources, particularly if overgrazed [2]. There are also direct emissions of different types of greenhouse gases from the livestock that use these areas. Understanding the dynamics of carbon cycling and greenhouse gas emissions in pastures is critical in limiting climate change [3].

The extent to which climate change may be mitigated by producing and consuming fewer livestock products (meat and dairy) is highly contested. Some disagreements reflect the presence of vested interests or participants with strong ideological standpoints, but there is also considerable uncertainty around the natural science evidence base of relevance to policymakers. An important question is the extent to which carbon can be sequestered in the soil of grasslands grazed by livestock. This is difficult to answer for several reasons. First, carbon sequestration and stocks are influenced by the chemical and physical make-up of the soil matrix, by the local environment and by how the land is managed [4]. The rate of carbon sequestration may also vary greatly over time. For example, rates can initially be very high on degraded soils that are depleted in soil carbon, but then decline as carbon builds up over time and ultimately reach an equilibrium. Second, in calculating the net benefits of pasture as carbon stores, account needs to be taken of the emissions associated with the livestock that feed on it, as well as further emissions in the meat and dairy food processing chains. This calculation is complicated by the fact that unlike many other sources of greenhouse gases, livestock production is associated with not only carbon dioxide emissions but also considerable amounts of methane and nitrous oxide [5,6]. In formulating policies, it is important to take into account the different dynamics of these gases in the atmosphere. Third, the counterfactual of how much carbon could be stored by the land if it was managed in a different way, for example if it was forested, is relevant to policy; as is the question of whether any advantage of reducing meat or dairy production in one area might be reduced or reversed by increased production elsewhere which may be more or less carbon emission intensive. The wide range of estimates in the peer-reviewed scientific literature of the amount of carbon stored in pasture soil reflect these complexities. Outside the scientific mainstream there are some remarkable claims about the extent to which anthropogenic carbon emissions may be mitigated by sequestration in pastures and rangeland that need to be subjected to critical scrutiny.

Here, we attempt to summarize the science evidence base concerning carbon storage in pasture land used for livestock production, in a policy-neutral manner that is accessible to policymakers who have some background in this area but are not subject specialists. The format of the review is a ‘Restatement’ where the summary is given as numbered paragraphs in an appendix. Because of the size and complexity of the topic, we focus our attention on carbon storage in pasture and grassland used for livestock production in the UK, though we hope the review will also be useful in other countries.

## 2. Material and methods

The relevant literature on grassland management, grazing livestock and soil carbon storage was reviewed with particular focus on studies in the UK and a first draft evidence summary produced by a subset of the authors. The statements and their assessments were subsequently debated via correspondence until a consensus was achieved. The near-final draft was then sent to a wide set of stakeholders (see Acknowledgements) for comments. We use the following restricted terms to describe the evidence, indicated by abbreviated codes, which are similar to those used in previous Restatements [7]. Codes at the end of paragraphs after full-stops indicate that they apply to the whole previous section; codes preceding full-stops or within a sentence apply to that sentence or clause alone.

[B] Uncontentious background material.

[S] Strongly supported by a substantial evidence base where further information is unlikely to change the current consensus.

[L] Less strongly supported by the existing evidence base and where further information might alter the current consensus.

[E] Expert opinion based on information from related sources or general principles from different fields of science.

## 3. Results

The summary of the natural science evidence base relevant to grassland management, grazing livestock and soil carbon storage policymaking in the UK is given in the appendix, with an extensive annotated bibliography provided as electronic supplementary material.

## 4. Discussion

We comment here on several general themes that emerged from our attempt to summarize a disparate and sometimes contradictory literature.

First, part of the disagreement about the extent to which carbon can be sequestered in grassland is due to different methodologies in measuring carbon in the soil. Efforts to provide standards and platforms to allow more meaningful comparisons are valuable and important. There are also differences in methodologies to assess the totality of emissions from a grassland production system, and to make valid comparisons with other more intensive ways to produce meat and dairy. Very different results are obtained if emissions are compared per hectare of land versus per kg of product. It is also not straightforward to integrate factors such as point-emissions from the manure produced in intensive systems. Finally, there are no agreed ways to account for the indirect effects of different production systems such as displaced or replaced production, even though ignoring these can greatly distort the climate impact of different policies. Progress in developing better, standardized carbon accounting tools, as well as accounting for indirect effects, would greatly assist policymakers.

Second, developing policy around carbon sequestration in grasslands inevitably has an important temporal component. Carbon build up in soils can be very rapid when heavily degraded arable soils are laid to grass but the rate of accumulation eventually approaches zero. Some of the most dramatic estimates of the carbon sequestration potential of grasslands in the advocacy space take such initial accumulation figures and assume they can be applied to all pastures and in perpetuity. Measures of total emissions from grassland have to grapple with the different dynamics of carbon dioxide and methane in the atmosphere. Carbon dioxide accumulates in the atmosphere causing progressive warming while methane, produced by ruminant enteric fermentation or methanogens in waterlogged organic soils, relatively quickly reaches an equilibrium concentration so that constant rates of emissions cause a fairly stable warming. Reducing emissions of any greenhouse gas can contribute to climate change mitigation, but the evidence base and the broader policy context must together determine which particular policies to consider and/or prioritize. For example, a broader policy focus might be the urgent need to prevent future warming in the next few decades to avoid potential Earth-system tipping points and give time for technological advances to help decarbonize other sectors. In that case, policymakers might place a high premium on measures to get carbon dioxide out of the atmosphere now (carbon capture in heavily degraded soils) and on reducing methane emissions (which acts to cool the environment). Landowners and farmers who make these land use changes or reduce livestock emissions are producing ‘public goods’ (benefits to society), an argument for public funding. A further complication is the possibility that limiting meat or dairy production in one place is compensated for by increased production elsewhere which may be less (or more) carbon efficient.

Third, though the evidence base is inevitably not as comprehensive as desirable, it does suggest some obvious no-regret policies. A variety of management practices on pasture, discussed in this Restatement, have been shown to have both environmental and economic benefits to the landowner. Many farmers and landowners are implementing them but more would be encouraged to if provided with better advice and guidance. A different type of policy where the evidence base is clear, concerns the importance of maintaining peatlands as carbon stores and ensuring that if they are used for grazing, livestock densities are kept below a level at which peat damage causes greenhouse gas emissions.

All high-income countries, to differing degrees, subsidize their agricultural and land sectors, in large part because their labour costs are higher than in lower-income countries. Subsidies may be explicit as in the area-related cash-transfers in the European Union's Common Agricultural Policy, or more indirect as in the United States' Farm Bills’ provisions which, for example, provides low-cost insurance against yield losses. Some will argue that it is economically inefficient to support a particular sector in this way, but the political reality is that these transfers will continue. Accepting this, there is the opportunity for the state to get more out of its investment. The challenge is to design the means to incentivize the provision of public goods in a way that is transparent, easily implementable, avoids major transaction costs and takes into account indirect effects. To do this successfully, policymakers need to be able to access summaries of the evidence base that are policy neutral in the sense of not being designed to support a particular advocacy position. A Restatement seeks to summarize the complex literature in an area in a way that is useful to policymakers who have knowledge of the issue but are not deep subject specialists.

## Figures and Tables

**Figure 1.**
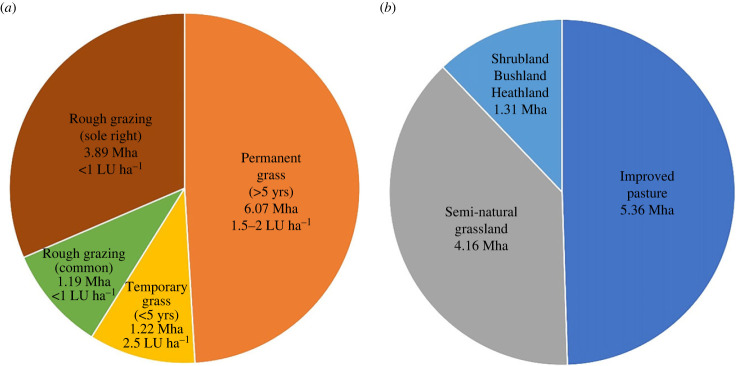
(*a*) UK agricultural area that is grassland or used for grazing, total 12.4 Mha (million hectares) (1). Typical stocking rates in livestock units per hectare (LU.ha−1) are also given, based on industry recommendations, where cattle over 2 years are 1 LU, and a lowland ewe with lamb is 0.12 LU. (*b*) UK land cover of habitat types that can be used for grazing (2).

**Figure 2.**
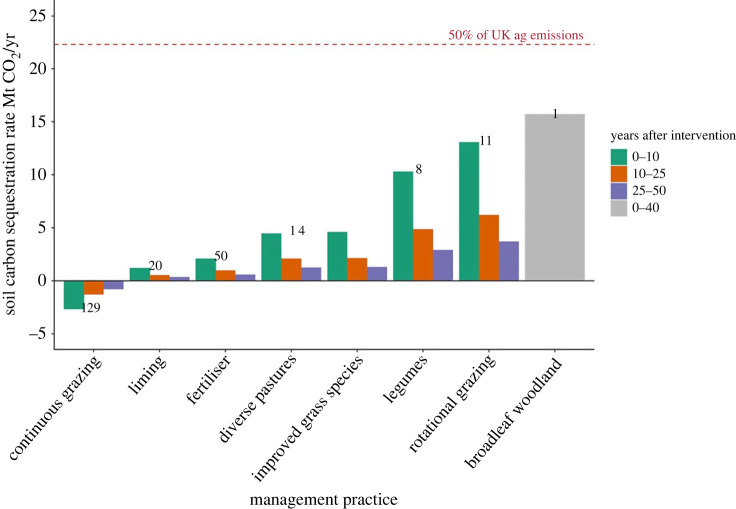
Carbon sequestration or emission rates from adopting grassland management practices, assumed to be adopted over 20% of UK improved pasture area from figure 1*b* (i.e. 1.07 M ha). Positive values are carbon sequestration, negative values represent emissions. Average sequestration rates presented from years 0–10, 10–25 and 25–50 following intervention adoption, demonstrating the declining rates of sequestration/emissions over time as soil carbon stocks approach a new equilibrium. The numbers above each group of bars indicate the number of primary studies underpinning that data point. The red dashed line at 22.3 Mt CO_2_e.yr represents half (50%) of UK agriculture emissions in 2020 (total 44.6 Mt CO_2_e (3)), included as a point of reference.

## Data Availability

The data are provided in the electronic supplementary material [8].

## Appendix A

**(a) Aims and scope**

(1) Substantial carbon is stored in pasture systems managed for livestock production. An important policy question is whether changes in land use and management practices can affect carbon sequestration in pastures and potentially contribute to climate change mitigation. Analyses of these issues have to consider the complete greenhouse gas budget of livestock production as well as the counterfactuals of using the land for other purposes.
(2) This ‘Restatement’ aims to summarize the science evidence base relevant to the development of policy around carbon sequestration in pastures used for livestock production. There is a focus on evidence of greatest relevance to the UK, where the climate, topography and soil types are particularly suited to pastoral agriculture.
(3) This Restatement is structured as follows. The greenhouse gas emissions from livestock production are described and summarized (section (b)) followed by a description of soil carbon dynamics (c). Estimates of carbon stocks in pastures and what they might be under alternative land uses is then summarized (d) with the next two sections examining the evidence for the influence of grazing management (e) and other management practices (f) on soil carbon. The last two sections detail indirect effects of pasture management decisions (g) and other considerations (h) of which policymakers should be aware.

**(b) Livestock emissions**

(4) The global food system is responsible for approximately 34% of all greenhouse emissions annually. Direct emissions from food production, land use change associated with agriculture and supply chain activities, each account for approximately a third of this total. Globally, meat and dairy production are responsible for around 57% of all food system emissions. [S]
  (a) In addition to carbon dioxide (CO_2_) emissions, livestock production leads to the emission of methane (CH_4_), a potent greenhouse gas but with a short average atmospheric residence time of about 9 years, and nitrous oxide (N_2_O), which is more potent than CH_4_ and has an average atmosphere residence time of approximately 115 years. CO_2_ persists for a very long time in the atmosphere, so from an emissions policy perspective, this gas can be treated as cumulative (see ¶46). [B]
  (b) Metrics have been developed to allow the effects on climate of emissions of different greenhouse gases to be compared and combined, typically reporting the warming effects of other greenhouse gases as ‘CO_2_ equivalents’ (CO_2_e). The most commonly used metric is the 100-year Global Warming Potential (GWP_100_) defined as the amount of CO_2_ that would result in the same marginal climate impact over a 100 year period post-emission. A criticism of GWP_100_ is that it does not well capture the different dynamics of short-lived and long-lived greenhouse gases. To address this, an alternative emission metric, GWP*, has been developed to reflect the fact that constant emissions of a short-lived gas lead to an equilibrium concentration of that gas in the atmosphere which makes a stable contribution to global warming, whereas constant emissions of a long-lived gas results in the gas accumulating in the atmosphere and having an increasing effect on temperatures. [B]
  (c) Livestock emissions are highly variable across geographical regions and production systems, meaning care must be taken when applying global-average figures to specific contexts [B]. For example, beef produced in western Europe generates only one-third of the average global emissions levels (CO_2_e per kg of carcass weight) [S].
(5) Livestock associated emissions in the UK mainly come from enteric fermentation, fertilizer manufacturing and application, manure storage and application, feed production, and the energy used in agriculture and the food supply chain. Globally, land conversion from natural or semi-natural vegetation to agriculture for livestock grazing or feed production is also a significant source of emissions. [B]
  (a) Ruminant animals (cattle, sheep, goats) use enteric fermentation to digest grass and other coarse plant material [B]. This produces CH_4_, which together with livestock manure, accounts for one-third of global anthropogenic CH_4_ emissions [S].
  (b) CH_4_ and N_2_O are emitted both when livestock excreta are deposited directly onto pastures, and when manure from indoor systems is stored and then spread onto fields as fertilizer. Emissions per unit of excreta produced are lower in pasture than in indoor systems, due to the fermentation dynamics of stored manure. Manufactured fertilizers applied to pasture and feed crops also lead to significant N_2_O emissions. Some nitrogen (N) from livestock excreta and fertilizers is also lost via leaching into water and volatization into the atmosphere as ammonia (NH_3_), followed by deposition onto land elsewhere, which both result in indirect N_2_O emissions. The production of manufactured fertilizers is energy intensive and a source of CO_2_. [B]
  (c) CO_2_ emissions arise from land use change, energy generation for on-farm machinery, supply chain transport, feed and food processing, and other on-farm and food-chain activities. [B]
    (I) Worldwide, deforestation and conversion of other habitats to create pasture and cropland to grow animal feed are responsible for approximately 10% of global livestock-associated emissions. [S]
(6) The greenhouse gas emissions per kg of meat or dairy vary with the production system and feed type. Globally, 65% of all livestock emissions at present come from beef and dairy cattle. [S]
  (a) There are approximately 9.6 million (M) cattle in the UK, with 1.5 and 1.9 M breeding females in the beef and dairy herds, respectively. The UK has 33 M sheep, of which 16 M are breeding females. Agriculture accounted for 11% of UK emissions in 2020 (reflecting food produced rather than consumed), of which 45% was due to enteric fermentation by sheep and cattle, and 12% due to livestock manure. [B]
  (b) For meat produced in the UK, typical greenhouse gas emissions are substantially lower than global averages. UK-specific emissions have been estimated at: beef, 11–25; lamb, 17; pork, 6.4 and chicken, 4.6. All figures given as kg CO_2_e per kg of carcass weight including bones from ‘cradle’ to farmgate. A litre of milk is responsible for 1.1 kg CO_2_e. [S]
    (I) Emissions are sometimes expressed per kg of edible protein or per total (or digestible) amino acids or micronutrient content [B]. Such metrics can be useful in comparing among substitutable food types, but selecting measures that favour particular food types has also been used for advocacy purposes [E].
(7) A number of different measures and interventions have been proposed or are being researched to reduce the direct greenhouse gas emissions from livestock. Improved animal husbandry and genetics can increase production efficiency leading to lower emissions per kg of meat or dairy produced, while livestock nutrition and feed supplements may reduce methane production.  [S]
  (a) CH_4_ is produced from fermenting cellulose-rich material, so increasing the digestibility and starch or fructan content of forage by manipulating plant species or variety composition, or plant age at time of grazing, can reduce emissions. Cereal-based and ‘concentrate’ feeds also lead to lower methane emissions. [S] Although, full life-cycle assessments are required to assess the overall benefits of different strategies [E].
  (b) Manipulation of rumen chemistry and microbiota can also reduce emissions.
    (I) Feed additives, such as nitrates and ionophores, inhibit methanogenic bacteria or provide alternative metabolic pathways to those ending with CH_4_. Feed additives can be difficult to deliver when livestock are not fed in confined systems. [L]
    (II) Feeding cattle diets rich in lipids, condensed tannins or seaweeds, particularly red algae, also reduces CH_4_ emissions. Condensed tannins are naturally available in some forage species such as sainfoin (*Onobrychis* sp.) and birdsfoot trefoil (*Lotus* sp*.*), as well as in willow trees (*Salix* sp*.*) which can be browsed by livestock in agroforestry systems. There is uncertainty about the overall emissions reduction potential in farm contexts, as well as possible animal welfare and health, and environmental impacts. For example, the bromoform and iodine contents of seaweed currently limit how much can be safely fed to animals, thus restricting their emissions reduction potential. [L]
  (c) Genetic breeding programmes can produce animals that grow faster, have greater muscle volume or milk yield, improved fertility and lower maintenance requirements. [B]
    (I) For example, the use of female-sexed semen in UK dairy cows has reduced the number of pure dairy calves required to breed replacement cows, enabling higher meat yield beef sires to be used on much of the herd, resulting in more meat produced for the same number of animals. [B]
    (II) Genetic breeding for increased efficiency may have negative welfare impacts or result in animals less suited to outdoor production systems; for example, by reducing robustness and resilience to variable environmental conditions. [B]
    (III) CH_4_ production in ruminant animals is a heritable trait (heritability of 0.14–0.26), mediated through host genomic influences on the rumen microbiome, suggesting the potential for breeding programmes to substantially reduce CH_4_ production over a small number of generations. [L]

**(c) Soil carbon dynamics**

(8) Carbon exists in the soil in two broad forms—organic and inorganic. Soil organic carbon (SOC) is derived from the remains of living organisms, while soil inorganic carbon (SIC) comes from carbonate rocks and minerals, such as chalk and limestone. Both components influence the global carbon cycle, but the dynamics of SOC are more rapid and more relevant to the degree to which soils can be managed to store and sequester carbon. [B]
(9) The existing level of SOC in the soil is referred to as the *stock*, and is typically measured in tonnes of carbon per hectare (t C ha^−1^). Any increase in soil carbon stocks is referred to as *sequestration*. Soil carbon sequestration achieves a net removal of CO_2_ from the atmosphere and is typically measured in tonnes of CO_2_-equivalent per hectare per year (t CO_2_e ha^−1^ yr^−1^). Maintaining existing soil carbon stocks is important to avoid releasing additional CO_2_ into the atmosphere, but only soil carbon sequestration removes CO_2_ from the atmosphere and hence contributes to climate change mitigation. [B]
(10) CO_2_ is absorbed from the atmosphere by photosynthesis in green plants. Stocks of SOC in the soil are increased by the addition of dead plant material from above-ground plant litter, growth of below-ground roots, root exudates from living plants and through the addition of any animal excreta containing partly digested plant material. Microorganisms in the soil, which make up a small (2–4%) but important living pool of SOC, use other SOC components for energy and growth, leading to CO_2_ release through respiration, while also making nutrients available to plants through mineralization. [S]
(11) The flow of carbon through different fractions of SOC is measured and modelled using different approaches. One widely adopted approach measures two broad pools of soil organic matter (SOM; approximately 50% carbon by weight and so proportional to SOC) that decompose at different rates. [B]
  (a) *Particulate organic matter* (POM) is relatively undecomposed material present in large particles (53–200 µm). POM can be decomposed quickly by soil microbes with some used for energy and released as CO_2_, and some used for growth (increasing microbial biomass). [S]
  (b) *Mineral associated organic matter* (MAOM) is strongly bound to small soil mineral particles (less than 53 µm), particularly clay minerals, and predominantly originates from plant root exudates and microbial residues. Because microbes are tiny and live on soil particles and in the small pores between them, when they die their residues can get stuck to the soil minerals. MAOM is important for soil structure because it helps to hold soil particles together. This fraction is therefore relatively persistent and less susceptible to microbial degradation. [S]
(12) The amount of SOC generally decreases with depth and is determined by how far plant carbon (as roots, root exudates or their breakdown products—POM, MAOM and dissolved organic carbon (DOC)) can move down the soil profile. Although the top soil layers contain more SOC than the subsoil, conditions in the subsoil slow down the breakdown of SOC. [B]
  (a) SOC in surface soil layers can be lost through wind or water erosion as fragments of partially broken-down plant material (POM) or bound to soil particles (MAOM), or by leaching of DOC out of the soil into waterways. This can be prevented through maintenance of plant ground cover and rooting structures which stabilize soils. [B]
(13) The difference between the rates of SOC addition and removal determine whether soils sequester or lose carbon, and an equilibrium SOC level occurs when the two rates are the same. Cropland under continuous arable cropping stores relatively less SOC at equilibrium compared with permanent pasture which stores much more (figure S1). Following a change in land use or management, soil carbon stocks can take decades to centuries to reach a new equilibrium (influenced by climate—fastest in the tropics, slower in boreal regions), but the most rapid SOC changes occur in the first 20–50 years (see figure 1 and figure S1). [S]
  (a) Depletion of SOC is used as an indicator of soil degradation. There is substantial potential for SOC to accumulate if management practices change, until a new higher equilibrium is reached. [S]
  (b) A related concept to soil carbon equilibrium is that of ‘sink saturation’, which occurs in mineral soils (on which the majority of livestock grazing occurs) once all available MAOM binding sites are occupied. At sink saturation, little further sequestration is possible, even if soil carbon inputs were to increase, limiting the impact of management interventions. [S]
    (I) Approximately 80% of European grassland topsoil is currently below this sink saturation point, suggesting a considerable scope to increase carbon stocks further through sequestration. [L]
  (c) In wet anoxic peaty soils and peat bogs, microbial activity is reduced and soils can continue to accumulate large amounts of carbon as partially decomposed plant material, albeit very slowly. [B]
(14) Soil carbon sequestration is reversible if organic inputs to the soil (i.e. plant residues and livestock excreta) are not maintained or if the rate of microbial degradation is increased, even after the SOC has reached sink saturation. [S]
  (a) Soil tillage, particularly of previously uncultivated soils such as long-term pasture, results in the rapid loss of soil carbon by exposing more organic matter to microbial degradation because it stimulates microbial activity by increasing soil oxygen levels and temperatures (see ¶15(d)). [S]
    (I) Where repeated tillage is replaced with no-till techniques in croplands, a redistribution of carbon within the soil rather than a substantial overall increase often occurs. Transitioning from repeated tillage to no-tillage reduces SOC losses, but where soil carbon inputs are limited (particularly in croplands where there are periods of bare soil between annual crops and limited photosynthetic activity relative to perennial systems), no-till alone is unlikely to meaningfully increase soil carbon stocks. [S]
(15) SOC formation and persistence are affected by soil characteristics which in turn are influenced by the parent material from which the soil has formed, as well as local physical conditions [B]. POM formation is mainly determined by soil temperature and moisture, although these factors separately influence plant productivity and therefore carbon inputs. N availability positively affects the formation of MAOM, while its amount is chiefly determined by soil clay content [S].
  (a) Clay soils accumulate larger quantities of MAOM than do sandy and silty soils because more mineral binding sites are available. [B]
  (b) Binding of SOC to minerals is inhibited in very acidic soils (pH < 4) [B], an effect that can be mitigated by adding lime [S].
  (c) If oxygen movement in the soil is reduced, due to compaction, limiting space between soil particles or because the soil is very wet, certain microbial activity and associated SOC loss is reduced. However, if excess N and carbon are available, N_2_O emissions are enhanced by anaerobically active microbes. [B]
    (I) Draining wet high-organic soils and peatlands can lead to major increases in greenhouse gas emissions through higher microbial decomposition. In addition, dry peat is susceptible to shrinkage and loss by wind erosion. [B]
  (d) Higher temperatures stimulate microbial activity and hence carbon loss from soils. This may be partly mitigated by higher plant productivity and hence greater potential carbon inputs into the soil, where plant growth is not water limited. [B]
    (I) In large-scale geographical comparisons, desert areas contain the smallest amounts of SOC, and boreal forests contain more SOC than tropical or temperate forests, per unit area. [S]
    (II) Although tropical forests contain the most carbon in plant biomass, total plant plus soil carbon stocks per hectare are largest in boreal regions. [S]
    (III) There is a concern that a warming world will increase carbon losses from soils, particularly from the POM fraction. The accumulation of dead microbes from a larger soil microbial population may partially offset this in soils where sufficient moisture, carbon and nutrients are available. Increased plant productivity due to the fertilization effect of increased atmospheric CO_2_ concentrations may also occur. [L]
  (e) Because MAOM levels are largely determined by microbial activity, the availability of nutrients for microbes limits the formation of SOC. Despite the importance of N availability in SOC formation, applying N through manufactured fertilizer is unlikely to be a good strategy for climate mitigation because the microbial reduction of available N to N_2_O can outweigh gains in soil carbon from increasing N availability. [S]
    (I) Poor soil organic matter levels in croplands limits organic N available to support crop productivity, so additions of manufactured N fertilizer may be warranted regardless of the lack of a direct beneficial effect on net emissions in order to increase yields, thus maintaining food production while reducing the overall land required for agriculture. [E]
  (f) The composition and diversity of soil microorganisms also influence the rates of SOC turnover and stabilization. Organic material from dead microbes is the source of around 50% of total SOC in grassland topsoil. Fungi play a greater role in SOC accumulation than bacteria, due to their close association with plant roots, greater carbon use efficiency and production of more stable types of organic matter. Fungal abundance is reduced in acidic soils (see ¶15(b)). [S]
(16) Soils can also be responsible for CH_4_ and N_2_O emissions. [B]
  (I) Soil CH_4_ emissions largely occur under anaerobic conditions. Some soil bacteria are methanotrophic and so can metabolize atmospheric CH_4_ (to CO_2_) but the net flux from normal aerobic soil is near zero. [S]
  (II) N_2_O emissions increase with the availability of excess mineral N in the soil and under anaerobic conditions. Excess soil mineral N from manufactured fertilizers, excreta and N-fixing plants such as legumes can increase N_2_O emissions from agricultural soils. [B]

**(d) Soil carbon in pastures and under alternative land uses**

(17) Global soils contain approximately 1400 Gt of carbon to 1 m depth, as compared with approximately 480 giga tonnes (Gt) in forest biomass and litter and approximately 890 Gt tonnes as CO_2_ in the atmosphere. Approximately 26% of the global land area is grassland (including rangelands, shrublands, permanent pasture and temporary pasture), which contains approximately 20% of soil carbon stocks. [S]
(18) 9.8 Gt of carbon are stored in soils in England, Wales and Scotland, 52% of which is in peatland and total greenhouse gas emissions in 2020 for England, Wales and Scotland was 0.103 Gt C-CO_2_e. Permanent and temporary grasslands cover half of the UK (approximately 12.4 Mha), and accounts for 72% of agricultural land. UK grasslands can be categorized by their intensities of agricultural use, soil and vegetation types and carbon stocks (figure 2). [S]
  (a) Permanent pasture, which may be semi-natural and used as ‘rough grazing’ or agriculturally ‘improved’ through the addition of forage species, lime or fertilizer, contains 1.2 Gt of carbon in the top 1 m of soil.
  (b) Temporary grassland (or leys which form part of an arable rotation) are typically included in SOC estimates for cropland (total approximately 0.7 Gt for GB).
  (c) Peatland covers 3 Mha, or 12% of the UK land area. Of this, 1.5 Mha is used for extensive grazing of livestock, predominantly on blanket bogs in the uplands, and a further 0.2 Mha for cropland in the lowlands. [S]
    (I) The total net emissions of UK peatland are approximately 0.006 Gt C-CO_2_e per year due to existing degradation. Of these emissions, 32% are from arable cropland on drained peat, and a further 27% from peatland converted to agriculturally improved pasture, predominantly in the lowlands, corresponding to 7 and 8% of UK peatland area, respectively. [S]
    (II) Peatland in near-natural condition is approximately carbon neutral. Restoration of peatland currently used for livestock grazing, such as by rewetting drained areas or reduced stocking rates to allow peat-forming vegetation to recover, has substantial long-term emissions mitigation potential, although there may be short-term net emissions increases when peatland is first re-wetted. [S]
(19) Tillage of permanent pasture for reseeding or conversion to cropland leads to substantial soil carbon loss. Tillage breaks apart soil aggregates, exposing organic matter to microbial degradation, which is stimulated by associated increases in soil temperature and available oxygen. Temporary grassland and croplands also have reduced soil carbon inputs leading to smaller equilibrium SOC stocks, in part because the plants they support have less well-developed root systems and, in croplands, because plants are typically present for only part of the year and there are periods of bare soil. Converting cropland or temporary pasture to permanent pasture increases soil carbon as these processes are reversed (figure S1). [S]
  (a) Reversion of cropland to grassland (grazed or otherwise) has been a major source of soil carbon sequestration in Europe between 1950 and 2010. [S]
(20) Introducing temporary grass-based leys into arable rotations leads to larger soil carbon stocks compared with continuous arable cropping but still much less than permanent pasture. [S]
  (a) Leys in arable systems also have other benefits including soil stabilization and reduced erosion because of plant roots (especially if they include deep-rooted species), and increased fertility where N-fixing plants are present. [S]
  (b) *Pasture cropping* involves sowing arable crops directly into pasture without tillage. This avoids significant losses of carbon, although leads to lower yields compared with no-till cropland. It is most feasible when crops that grow in the cool season are sown in pastures containing grasses that only grow in the warm season, so that competition with the crop is reduced. A practice more applicable to the UK is to *undersow* arable crops with pasture species, to avoid a period of bare ground after the crop is harvested and before a temporary ley is established, and which can maintain or even increase arable yields if an appropriate species combination is chosen. [L]
(21) Conversion of grassland to woodland typically increases total carbon stocks because, although soil stocks are typically similar, much more carbon is stored in woodland as above-ground biomass. The net change in carbon stocks is influenced by underlying soil type and depth and the form of afforestation. [B]
  (a) On mineral soils with low organic content, typical of temporary pasture and cropland, woodland creation tends to increase soil carbon stocks as woodland soils contain more POM [S]. If tree planting involves major soil disturbance, it can take 5–10 years for net carbon sequestration to occur (including tree biomass) and over 20 years for the soil carbon stocks to be restored [E].
  (b) On soils with high organic content such as shallow peats and organo-mineral soils, more soil carbon is lost during tree planting, so net sequestration and build-up of carbon stocks is delayed by several decades. [S]
  (c) Planting trees on UK deep peat soils, now prohibited, requires drainage and results in lowering of the water table with subsequent substantial carbon losses. [S]
  (d) Broadleaf trees are more likely than conifers to maintain or enhance both soil carbon stocks and biodiversity if planted on temperate pastures. [S]
  (e) Planting methods that reduce soil disturbance or that allow natural regeneration to occur, including conservation grazing or rewilding approaches, reduce carbon loss from the soil. Natural approaches result in slower woodland establishment and carbon accumulation, although the intermediate scrub habitats have biodiversity benefits. [E]
(22) Silvopasture, a form of agroforestry, integrates trees into livestock pastures, either on field boundaries or as rows or clumps within the field [B]. It entails limited soil disturbance compared with woodland establishment [L] which protects below-ground carbon stocks, while later leaf deposition and above-ground biomass accumulation adds to carbon stocks [S]. Livestock grazing densities may be lower in mature silvopasture although individual animal productivity can be higher due to reduced heat- and cold stress, but the carbon consequences of displaced production needs also to be considered [L].
  (a) Similarly, hedgerows can be incorporated or reintroduced into farming landscapes with minimal loss of agricultural area. Total carbon storage in hedgerows is influenced by their height and width, and whether broadleaf trees are allowed to mature at intervals, which has little additional impact on agricultural land area but positive carbon benefits. Soil carbon stocks under and in the immediate proximity to hedgerows are also increased by dead wood and litter fall. [S]
  (b) It is possible to achieve greater carbon stocks *per tree* by planting them in silvopasture rather than in a woodland, due to preserved pasture soil carbon stocks plus additional tree biomass. However, the carbon stock *per hectare* is greatest in woodland. [L]
(23) The same factors that influence carbon dynamics when converting grasslands to woodland also operate when grasslands are converted to permanent bioenergy crops, such as the grass *Miscanthus* and short-rotation willow or poplar. Like commercial forestry, the above-ground biomass is harvested rather than accumulating *in situ*. The consequences for carbon budgets then depend on whether the embedded carbon re-enters the atmosphere, either rapidly via burning or more slowly via decomposition, or is captured and stored, in addition to the energy source it replaces. It is important to consider indirect effects, such as the opportunity costs of not using the land for another purpose or the alternative fuel sources displaced by biomass, when calculating overall carbon budgets. [B]

**(e) Grazing management and soil carbon**

(24) The presence of livestock, and how livestock are managed, have multiple effects on soil carbon dynamics and storage, as well as on plant net primary productivity (NPP), biomass removal and livestock emissions. Plant productivity in turn underpins the CO_2_ flux of soils. Livestock excreta affects soil nutrient availability, while animal trampling changes the physical properties of soil such as its density. Grazing management also influences forage quality (proportion of vegetative versus senesced plant material), which in turn impacts the level of ruminant enteric methane emissions (see ¶5(a)). [B]
  (a) The scientific evidence base for the effects of different pasture management regimes on carbon stocks and flows is relatively limited. Medium- and long-term studies at field scales would be of great value to policymakers. [E]
(25) Continuous grazing frequently leads to net removal of vegetation biomass. Much of the embodied carbon is released as CO_2_ through animal respiration or CH_4_ through enteric fermentation and frequently results in reduced organic matter entering the soil. Meta-analyses show that continuous grazing reduces soil carbon by 3–31% compared with grazing exclusion in moist cool climates such as the UK, with greater reductions at higher grazing intensities [S]
  (a) In some circumstances, continuous grazing can increase plant productivity and therefore soil carbon inputs.
    (I) Moderate grazing of low-productivity semi-natural grasslands that have evolved under intermittent grazing pressure increases soil carbon stocks [S]. This is unlikely to directly apply to UK grasslands, due to their different evolutionary history and higher productivity [E].
    (II) In warmer and drier climates than the UK, continuous grazing, particularly at low intensities, can increase soil carbon. [L]
    (III) Overgrazing occurs when the rate of biomass removal by livestock exceeds the capacity of the forage to recover, and typically compromises plant productivity, livestock performance and soil carbon stocks [B]. Continuous grazing, also known as set stocking, is possible without overgrazing, although this requires careful pasture management with regular assessments of forage growth and adjustments of stocking rates [E].
(26) Rotational, as opposed to continuous, grazing is the practice of moving livestock around subunits of pasture to create alternating periods of grazing and no grazing [B]. When well-managed, these respites from grazing allows vegetation to regrow, which can lead to an overall rise in NPP with increased root development and exudates thus maintaining or increasing SOC. [L]
  (a) One meta-analysis suggested that rotational grazing could increase SOC by 21% in the first 3 years of implementation compared with grazing exclusion (although only the top 5 cm of soil was considered in this analysis) [L]. This rate of sequestration will decrease after 5–20 years as SOC stocks approach a new equilibrium, and the overall SOC increase will depend on soil type, particularly clay content, and initial condition, as degraded soils have higher capacity for sequestration [B].
  (b) A subset of rotational practices grazes high densities of livestock for short periods of time with long rest and recovery intervals; this can be called mob, tall grass, holistic planned or adaptive multi-paddock grazing. Grazing in this way can result in some plant foliage being trampled down rather than eaten, creating a layer of decomposing vegetation at the soil surface which can increase soil organic matter. [L]
  (c) The increased NPP in rotationally grazed systems can allow higher stocking rates which may lead to increased direct emissions, but possible reduced indirect emissions from displaced production. Alternatively, it can allow for a reduction in inputs (fertilizer, feed), which decreases direct and indirect emissions associated with inputs. [B]
    (I) Different management objectives can result in differing outcomes from adopting rotational grazing approaches. For example, a focus on livestock performance prioritizes maintaining forage in a highly palatable vegetative stage, and typically utilizes shorter rotations to prevent forage senescing. Other systems prioritize improvements in soil health and balancing the energy to fibre ratio in livestock diet for optimum ruminant functioning. In these systems, longer recovery periods without grazing are used to maximize forage accumulation, root growth and forage is frequently allowed to reach maturity before grazing. The precise soil-plant-livestock emissions balance across such variations in management regimes are highly context specific and have not been well quantified. [E]
  (d) The impact of rotational grazing depends on environmental conditions (temperature, rainfall, soil type) being suitable for vegetation regrowth between grazing episodes and this may limit its benefits. [B]
    (I) By contrast, adaptive and holistic grazing approaches aim to respond to changing environmental conditions. For example, areas of pasture can be removed from or added to the grazing rotation to match rapid versus slow rates of forage growth, respectively, at different points in the grazing season. However, ‘stockpiling’ forage biomass in this way can result in a trade-off with forage digestibility and therefore livestock performance and hence emissions per unit of meat or dairy output. [E]
  (e) Although well-managed rotational grazing approaches can increase soil carbon and agricultural productivity, the extent and magnitude of possible benefits is contested. [E]
    (I) Bold claims have been made by some advocates that approaches such as Holistic Planned Grazing could reverse desertification and fully mitigate anthropogenic climate change. However, the proposed mechanisms underpinning these assertions lack an evidence base [S], and the scale of the claims are implausible [E].
    (II) It has been suggested that adaptive, holistic or mob grazing approaches could help form ‘new’ soil and there is some evidence for increases in topsoil depth. True soil creation occurs very slowly and requires bedrock weathering and mineralization, and the observed increases in topsoil depth are likely to result from: (a) increase in the fine root mat typical of grasses at the soil surface and deeper roots increasing organic inputs to the subsoil, (b) partially decomposed plant litter accumulating on the soil surface and (c) improved soil particle aggregation and soil porosity reducing soil density for an equivalent mass. It is implausible that perpetually high rates of soil carbon sequestration will occur due to the saturation point of MAOM eventually being reached. [E]
(27) Allowing livestock to graze temporary pasture in ley-arable rotations leads to a greater increase (2–20%) in SOC than if grass is cut and removed [L]. Livestock excreta also increase nutrient availability for subsequent crops and may benefit soil biodiversity, although the direct and indirect emissions associated with livestock must be considered [S]. Livestock can also be used to graze over-winter cover crops in arable rotations, although there is currently insufficient evidence to determine the impact of this on soil carbon stocks [E].
(28) In assessing the net benefits of soil carbon sequestration due to grazing, it is important to include the direct and indirect greenhouse gas emissions from the livestock involved. [B]
  (a) Where soils are initially degraded, improved livestock management, such as reducing grazing intensity when overgrazing has occurred (¶26b), adopting rotational rather than continuous grazing (¶26) or integrating livestock into arable systems, can lead to an increase in soil carbon stocks particularly over the short term. Understanding the full carbon-budget implications requires considering system productivity and any indirect effects of higher or lower outputs. [E]
  (b) If introducing livestock into ley-arable rotations results in an overall increase in their numbers, then the overall carbon-budget effect is likely to be negative. But if livestock numbers do not change, positive outcomes may occur, for example because: (i) the temporary pasture that is used for grazing or forage production replaces other feed sources and their associated emissions, (ii) land is released for targeted sequestration projects, (iii) inorganic fertilizer and herbicide use is reduced on cropland due to livestock manure and grazing, respectively. Such seasonal reallocation of existing livestock from pasture areas to temporarily graze cropland is gaining traction with UK practitioners, but is limited by the spatial separation of the main cropland and livestock areas in the UK. [E]
  (c) In calculating the net climate benefits of altering grazing patterns to promote carbon sequestration, using the GWP_100_ CO_2_e metric may weight too highly the negative effects of CH_4_ emission (if livestock numbers do not change there will be no net increased warming through this route). Using the alternative GWP* metric allows the direct effects of changes in CO_2_ emissions and sequestration, and of changes in CH_4_ emissions, on climate warming over different time scales to be better integrated (see also ¶(47)). [S]
(29) Relatively low intensity ‘conservation grazing’ by domestic livestock at appropriate times can be an important management tool in maintaining a variety of semi-natural habitats of high biodiversity value (figure 1*b*), in some cases substituting for wild herbivores that are locally absent or extinct [B]. Because of their low stocking rate, the effects on soil carbon dynamics and net emissions are small [E].
  (a) Some semi-natural habitats are very sensitive to the presence of domestic livestock. For example, upland peatbog ecosystems can be damaged by densities of sheep above 1 per 2.5 hectares, which is the typical stocking density in these environments. Peat-forming vegetation is especially susceptible to trampling although limited grazing can still be desirable. [L]

**(f) Pasture management and soil carbon**

(30) There are multiple ways to impact pasture soil carbon dynamics, some via grazing livestock, and some by utilizing other interventions. This section explores the latter and their interaction with grazing interventions.
(31) Cutting or mowing grasslands with removal of biomass may reduce organic inputs to soil, but can also stimulate plant regrowth and therefore NPP, thus increasing root exudates and turnover which can lead to increased soil carbon. A recent meta-analysis indicated that grassland mowing with no biomass removal has no overall effect on SOC, although there is some evidence that increasing cutting frequency can result in SOC increase. [L]
(32) Low productivity ‘weedy’ grass species tend to accumulate over time in old intensively managed pastures, particularly if optimal soil pH for grass growth is not maintained. Pasture productivity and quality of forage for livestock production can be improved by reseeding with higher-quality species, including legumes (see (¶33)) and deep-rooted herbs (see (¶34)) [S]. Pasture rejuvenation can increase SOC by 1–2% per annum [L], particularly where seeding techniques are used that avoid tillage, such as direct drilling or ‘overseeding’, thus preventing soil carbon losses (see ¶(19)) [B].
(33) Introducing N-fixing legumes (clovers, trefoils, vetches) can increase plant productivity and SOC, as well as improve forage quality thus benefiting livestock productivity [S]. Inclusion of legumes increases N_2_O emissions due to higher soil N availability, which typically negates about 30% of the benefits of increased carbon sequestration in western European contexts, although legumes also influence soil structure and microbial activity which may lead to proportionally lower N_2_O emissions for a given N input [L]. However, where legume N-fixation replaces applications of manufactured N fertilizer, this both avoids the emissions from fertilizer manufacturing and reduces soil N_2_O emissions for similar forage dry matter production [B].
(34) Plants with deep roots, both herbs and grasses, are proposed to improve SOC stocks by delivering root exudates and dead organic matter to deeper soil horizons, as well as stimulating microbial activity at lower depths, although there is currently limited evidence from temperate regions to support this [E]. Deep roots can have other benefits such as improving drought resilience and facilitating water infiltration by increasing soil porosity which reduces runoff [L].
(35) Pastures with a high diversity of plant species, cultivars and functional groups can increase biomass production by around 30% and soil carbon stocks by approximately 18% [S]. High plant diversity can also increase mineral supply to livestock improving animal health and productivity. This happens because a diverse set of species can use available resources more efficiently and are affected in different ways by environmental stress thus conferring resilience. [E]
(36) The application of manufactured fertilizer frequently improves productivity and can increase SOC by around 10%, although pasture diversity is reduced due to fertilizer favouring the most competitive species. However, any increase in carbon sequestration from N fertilizer is more than outweighed by the increased N_2_O emissions after application, plus the emissions involved in its manufacture (via the Haber–Bosch process, responsible for 1.4% of global emissions). [S]
(37) The application of farmyard manure and slurry is a source of N and other key macro- and micronutrients, and contains organic matter that can be incorporated into the soil, thus increasing soil carbon stocks. This increase must be set against CH_4_ and N_2_O emissions during storage and application, especially in wet conditions. [S]
  (a) Long-term experiments in the UK indicate that there is little SOC benefit from applying manure to permanent pastures where these soils are already carbon saturated [L]. By contrast, applications on cropland increase soil carbon stocks by 20–30% [S].
    (I) In the UK, manure is increasingly being returned to cropland from livestock farms, often in exchange for straw for animal bedding, as arable farmers seek to restore cropland soil organic matter levels and reduce manufactured fertilizer usage. However, associated machinery compaction needs to be managed to avoid negatively impacting crop productivity or soil emissions, and the geographical separation of the main cropland and livestock regions currently limit uptake of this practice. [E]
  (b) Using manure applications to increase SOC stocks can risk simply redistributing organic matter, rather than leading to an overall increase. This is because if the components of manure, particularly crop residue used for bedding, were left on the field where they were grown, this would have increased soil organic matter in those locations anyway. [B]
  (c) Options to reduce emissions from manure include treatments such as anaerobic digestion or aerobic composting, storage under cover and reducing storage time, and application methods such as sub-surface injection.
    (I) Anaerobic digestion of manure and slurry often reduces CH_4_ emissions from storage, although emissions can be higher if digested manure is stored in warm conditions over summer, but may increase N_2_O emissions after application by making the N present more readily available to microbes. The overall reduction in emissions from manure storage and application due to anaerobic digestion ranges from approximately 20–60%. Fossil fuel usage can be displaced with the CH_4_ captured via the digestion process. Aerobic composting and aeration of slurry similarly reduces CH_4_ emissions which more than outweighs any increase in N_2_O emissions in CO_2_e. Treatment of manure with acids reduces ammonia, but may increase N_2_O emissions after application due to higher N retention and could increase field liming requirements to maintain optimal soil pH for plant growth. [L]
    (II) Injection of slurry just below the soil surface can reduce ammonia by 85% compared with conventional splash plate slurry application. The improved soil N retention reduces manufactured fertilizer requirements but N_2_O emissions are correspondingly increased. [L]
    (III) Modifying livestock diets, such as by reducing or changing the form of protein included, improving dietary protein:energy balance, and altering the way in which N is excreted can also reduce N in manure by around 20%, which reduces N_2_O emissions due to the lower availability of N. [L].
(38) Ground limestone can be applied to acidic soils to increase pasture productivity and improve soil structure (liming) [B]. Soil carbon sequestration is inhibited in highly acidic soils (pH < 4) and liming can increase SOC by around 6% [L].
  (a) Liming reduces N_2_O and CH_4_ emissions [L]. However, the net impact on greenhouse gas emissions varies between studies, due to a simultaneous increase in soil CO_2_ emissions from increased microbial activity as soil acidity is neutralized. Emissions associated with the production, transport and application of lime also need to be accounted for.

**(g) Indirect effects**

(39) The creation or destruction of pastures, and how they are managed, affects greenhouse gas fluxes and the amount of carbon stored above and below ground. In estimating the overall benefits or downsides of these changes, it is essential to consider any indirect changes to emissions and storage elsewhere. [B]
(40) Conversion of cropland to pasture will result in reduced crop production and increased or redistributed ruminant livestock production. However, where arable soils are significantly degraded, temporary grass leys can recover soil organic matter and fertility thus enabling a return to more productive arable cropping on rotation (see ¶20 and ¶27). Changing management practices to increase soil carbon stores may affect food production, positively or negatively. Reduced production in one place will tend to stimulate more production elsewhere, in the absence of policies to influence consumption practices. [E]
  (a) It is seldom possible to identify precisely where and how compensatory responses take place and instead they must be estimated by food system models that take into account how prices and demand are affected by changes in supply, and which incorporate realistic assumptions about within-nation and international trade. [E]
  (b) Deforestation, especially in the tropics, is a major source of greenhouse gas emissions, biodiversity loss and soil degradation, so where compensatory production to reduced UK outputs involves clearing tropical forests, the net effects on climate and other environmental outcomes are likely to be very unfavourable. [S]
(41) There is substantial variation in the emissions produced from different beef production systems. Grass-fed systems typically incur higher CH_4_ emissions and require more total land than grain-fed systems, although less cropland. N_2_O and CO_2_ emissions are normally higher in grain-fed systems. There are not major differences in emissions per kg of animal liveweight or meat between the two production systems, though a great range of values within each. [S]
  (a) Limiting livestock production to grass-based ruminants, known as ‘livestock on leftovers', would reduce the amount of cropland needed to produce feed and avoid competition between human food versus animal feed production. It cannot be applied to egg, pork and chicken production because pig and poultry production systems rely on cereal-based feeds, though they can utilize food waste. Though grass-fed ruminants utilize pastures unsuitable for arable crops the counterfactual of using the land for other purposes such as woodland creation for carbon sequestration or for biodiversity should be considered. [S]

**(h) Policy implications**

(42) Many grasslands around the world are severely degraded and better management, particularly reductions in overgrazing, could lead to the sequestration of 1.65 Gt CO_2_e yr^−1^ globally (approximately 3% of annual global emissions) [S]. There is some opportunity for increasing carbon sequestration in grassland soils degraded from overgrazing and tillage in the UK, although well-managed permanent pastures that are already high in soil organic matter will have limited capacity for further sequestration though if maintained in good condition they can act as substantial carbon stores [E].
  (a) The overall technical potential for soil carbon sequestration from appropriate pasture management in the UK has been estimated at 2.95 Mt CO_2_e per year (approximately 0.7% of UK current emissions), informed over 20 years. [L]
(43) The permanence of carbon storage needs to be considered when developing land use policy to increase carbon sequestration. Much carbon in soils, especially the POM fraction, can be quickly lost if appropriate management is not continued or if the land is ploughed or severely disturbed [S].
  (a) It will be important to explore the resilience of soil carbon stores to climate change. For example, changes in temperature and moisture will influence soil carbon dynamics (see ¶(25)), while extreme weather events can affect soil microbial and plant communities and lead to episodes of soil loss and erosion. Climate change may also have an indirect effect through its influence on forage production and livestock husbandry. [E]
(44) Ruminant livestock production systems in the UK are predominantly pasture based with little recent history of land use change and only moderate use of imported feed crops, meaning direct emissions from land use change are much less than the global average. UK and Eire CH_4_ emissions, per kg unit output, are also well below global average, due to existing high productivity and efficiency, and a suitable climate. [E]
  (a) A reduction in UK livestock production could have net negative climate implications if the displaced production was made up from less carbon efficient production systems elsewhere (and for the same reason increased production might be positive). Whether this occurs depends on the details of trade networks and different product substitutability. [E]
  (b) For similar reasons a switch from more animal- to more plant-based diets in the UK, which would reduce emissions, need not imply a corresponding fall in livestock production if UK livestock products are competitive on global markets, especially were carbon trading to be widely introduced in the food sector. [E]
(45) Greenhouse gas emissions from the livestock sector can be reduced by supply-side measures (¶7), but for nations and the world to halt global warming demand-side interventions such as constraining global *per capita* meat consumption and reducing food loss and waste are unavoidable. [B]
  (a) People can be encouraged to reduce their consumption of high-emissions food types by education, persuasion, fiscal measures and regulation. The effect of the change depends on the quantity and emission intensity of the foods that they switch to. On average, appropriate plant-based substitutes such as pulses have substantially lower emissions than animal-based foods and so policies to reduce demand for meat and dairy would result in lower emissions. [S]
  (b) Some groups in low-income countries rely on animal-based products for their nutrition and should not be subject to meat consumption reduction policies in the absence of alternatives. The availability of minerals and vitamins differs between animal- and plant-sourced food which needs to be considered in formulating population nutrition policy. [E]
(46) As stated above (¶ 4(b)28(c)) emissions policies around livestock production need to take into account the different residence times of CH_4_, N_2_O and CO_2_ in the atmosphere. [E]
  (a) To stop further temperature increases, the world must achieve net zero CO_2_ emissions, but only prevent greater than present emissions of agricultural CH_4_. If livestock production were to remain constant with the same emissions intensity then its CH_4_ emissions would not contribute substantially to further global temperature increases beyond the amount of warming already caused by livestock CH_4_ emissions, though any CO_2_ and N_2_O emissions would. [E]
  (b) A sustained reduction in livestock production today would reduce the equilibrium concentration of CH_4_ in the atmosphere which would reduce the level of temperature increase from livestock. By contrast, the concomitant reduction in CO_2_ emissions would only limit further warming. [E]
  (c) Different metrics (GWP_100_, GWP*) report the warming effects of CO_2_, CH_4_ and other gases in different ways, and the most suitable metric is influenced by the precise policy goal it is intended to support. The widely used GWP_100_ can overestimate the long-term advantages of reducing CH_4_ emissions and underestimate the short-term benefits, an issue that GWP* or climate modelling approaches can address. [E]
(47) There are always multiple objectives when managing landscapes and grasslands, and these need to be considered in decisions around increasing carbon sequestration. For example, well-managed grasslands can improve water quality and reduce flood hazard. Both globally and in the UK, grasslands used as pasture produce food and support livestock farmers and allied rural businesses, and pastoralists with strong heritage and cultural value. Some grasslands also have important biodiversity value, and species-rich grasslands are threatened in the UK, although these frequently require specific management interventions that reduce their value for livestock production. [S]
  (a) Climate change, changing weather patterns and extreme weather events are likely to impact the ecosystem service of temperate grasslands. For example, changes in temperature and moisture will influence soil carbon dynamics (¶15(b-c)) and the resilience of forage productivity, while extreme weather events can drive shifts in soil microbial and plant communities.
